# Predictors of trajectories of child neurodevelopment in the first 2 years of life in LMICs: A systematic review and meta-analysis protocol

**DOI:** 10.21203/rs.3.rs-5285073/v1

**Published:** 2025-04-16

**Authors:** Mary Nyakato, Noeline Nakasujja, Richard Idro, Dickens Akena, Shubaya Kasule Naggayi, Andrew Sentoogo Ssemata, Anne Jacqueline Nakitende, Betty Nyangoma, Simple Ouma, John Mbaziira Ssenkusu, John.C Chandy, Paul Bangirana

**Affiliations:** Makerere University College of Health Sciences; Makerere University College of Health Sciences; Makerere University College of Health Sciences; Makerere University College of Health Sciences; Makerere University College of Health Sciences; MRC/UVRI and LSHTM Uganda Research Unit; Makerere University College of Health Sciences; Makerere University-John Hopkins University Research Collaboration: MU-JHU Care Limited; Global Health Uganda; Makerere University College of Health Sciences; Indiana University School of Medicine; Makerere University College of Health Sciences

**Keywords:** Predictors, trajectories, neurodevelopment, children, first 2 years, LMICs

## Abstract

**Background:**

In low-and-middle-income countries (LMICs), children are exposed to multiple risks that may compromise their neurodevelopment, especially during the early years. Early childhood developmental trajectories are crucial, especially in such at-risk populations as they help predict future neurocognitive potential. In LMICs where numerous factors shape child neurodevelopment, describing neurodevelopment trajectories and understanding the predictors that shape them is imperative for early intervention. The systematic review and meta-analysis will determine the predictors of trajectories of child neurodevelopment during the first 2 years of life in LMICs.

**Methods and analysis:**

The Preferred Reporting Items for Systematic Review and meta-analysis protocols (PRISMA-P) guidelines will be followed while performing this review. PubMed, Psych INFO, EMBASE, and Google Scholar databases and reference lists of relevant articles will be searched for articles. Selected publications will be uploaded to Endnote to remove duplicates and reviewed by title, abstract, and full text to identify those meeting the eligibility criteria. Longitudinal studies on child neurodevelopment and associated predictors among children aged ≤ 24 months in LMICs will be included. Screening, data extraction, and critical appraisal will be done by two autonomous reviewers. The Grading of Recommendations Assessment, Development, and Evaluation (GRADE) will evaluate the risk of bias and funnel plot asymmetry, publication bias. The I^*2*^ statistics will be used to test for heterogeneity in the selected studies and STATA-18 and EPPI-reviewer software for statistical analysis. A random-effects meta-analysis will be undertaken.

**Discussion:**

The protocol describes a systematic review and meta-analysis aimed at identifying factors influencing neurodevelopment trajectories during the first 2 years of life in LMICs. The review findings may provide a comprehensive understanding of the factors that influence child neurodevelopment, particularly in the first 2 years of life in LMICs, help identify critical windows of opportunity for intervention, and potentially guide the design of age and contextually appropriate interventions for optimizing neurodevelopmental outcomes, especially in this context.

**Systematic review registration:**

International Prospective Register of Systematic Reviews (PROSPERO), CRD42023421753.

## Background

Child neurodevelopment is essential as it lays the foundation for a child’s learning, cognitive, socio-emotional, and physical health, and well-being throughout life. Regardless, it remains an unmet priority, especially in LMICs where millions of young children are not achieving their full neurodevelopment potential due to adversity and exposure to multiple and cumulative risks [1, 2]. Adversities and risk factors especially early in life weaken this foundation as they disrupt typical neurodevelopment trajectories and may lead to an increased prevalence of developmental delay and deficits consequently affecting outcomes later in life such as school performance, health, and income in adulthood [3, 4].

In LMICs, factors that influence child neurodevelopment are multifactorial and interact in complex ways making it imperative to evaluate and monitor child development in this region and other at-risk populations [5]. Consequently, to truly understand early cognitive development, longitudinal studies are essential [6] as they provide an exceptionally rich evidence base for identifying the dynamic process of cognitive change, description of trajectories of development, and understanding of the effects of early childhood experiences and development over time [7].

Over the years, longitudinal and cohort studies have increased in LMICs, showing the complex interactions between risk factors and early childhood development [8]. Findings from the longitudinal studies conducted in LMICs have demonstrated that trajectories of neurodevelopment in this setting differ in number and trends. For instance, studies have demonstrated the detrimental impact of risk exposure on child neurodevelopment over time, evidenced by a reduction in neurodevelopmental scores [9, 10]. Similarly, findings from studies conducted among healthy children in the same region have reported decreasing neurodevelopment test performance scores with increasing age, especially between the first and second year of life [11, 12]. However, other studies have reported consistently increasing test scores over time [13]. A study conducted in 8 LMICs that assessed cognitive development at 6-, 15-, 24-, and 60- months of child age identified 5 trajectories of cognitive development: children with consistently high scores throughout, children with increasing scores, children with intermediate scores that had early decline or late decline and children with consistently low scores [14].

To improve child neurodevelopment in LMICs, it is crucial to understand neurodevelopmental trajectories and the predictors that influence these neurodevelopmental trajectories over time [15] Some studies have employed a longitudinal design to identify these factors [12, 14]. The study by McCormick and colleagues reported different variables to best discriminate between the trajectories of development at varying ages. For example, factors identified at 24 and 60 months of age include stimulation, how safe the environment at the child’s home was, and how responsive the caregiver was, both verbally and emotionally. At 0–24 months of age, the average zinc nutrient density in the child’s diet, the number of days the child experienced acute lower respiratory diarrhea infection, diarrhea fever, and vomiting, and the mother’s cognitive ability. Phytate and total energy from complementary foods were implicated at 9–24 months [14]. However, in another study conducted in India, socio-economic status and blood iron status were reported as other factors influencing change in cognition and motor scores respectively [12].

Regardless, data on trajectories of child neurodevelopment and factors that shape these trajectories, especially in the first 2 years of life in LMICs have not been synthesized. We will thus synthesize evidence on trajectories of early child neurodevelopment in LMICs and associated predictors. The review will not only provide a holistic view of child neurodevelopment over the first 2 years of life. Still, they may also inform the design of targeted interventions and policies that can optimize neurodevelopment in this region and other contexts where resources may be constrained.

### Rationale

This study aims to identify 2-year neurodevelopment trajectories from 0–24 months and examine factors associated with these trajectories. Understanding which specific factors have an impact on early development can inform further contextual/region-specific interventions aimed at alleviating the burden of compromised neurodevelopment, thus improving the lives of children living in LMICs, and bridging the development gap between children in LMICs and those in western high-income countries.

## Methods

### Design:

Systematic review and meta-analysis.

### Development and registration of the protocol

The systematic review protocol has been developed per the Preferred Reporting Items for Systematic Reviews and Meta-Analysis (PRISMA) guidelines. The review will be reported following the PRISMA-P 2015 statement, attached as additional file 1. The protocol was registered with the International Prospective Register of Systematic Reviews (PROSPERO), registration number CRD42023421753.

### Operationalization of key concepts

#### Neurodevelopmental trajectory

A neurodevelopmental trajectory will be defined as assessing neurodevelopment using 2 or more waves of data.

#### Low- and middle-income countries

Low-income economies are described as those that had a gross national income (GNI) per capita of $ 1,135 or less in 2022; lower-middle-income economies as those with GNI per capita between $1,136 to $4,465 and upper-middle-income economies with a GNI per capita between $4,466 to $13, 845 (https://blogs.worldbank.org/en/opendata/world-bank-country-classications-by-income-level-for-2024-2025). A comprehensive list of LMICs that will be considered in this review is attached (additional file 2).

#### Risk of Bias (RoB)

The risk of bias is defined as the likelihood that the study design used or how the study is conducted will give misleading results [16].

### Objectives

- To describe the trajectories of child neurodevelopment across the first 2 years of life among children in LMICs.
- To assess predictors of child neurodevelopment trajectories during the first 2 years of life among children in LMICs.

### Eligibility Criteria

We will include longitudinal quantitative studies that have utilized standardized tools to assess neurodevelopmental outcomes among children in LMICs. Additionally, the studies should have reported variables that affect neurodevelopment. We will screen articles for inclusion in the review and those selected/included will have to meet the following eligibility criteria.

### Inclusion criteria are studies that:

- Reported longitudinal neurodevelopment data (cognitive, motor, and language scores) at two or more time points. Longitudinal data will enable us to evaluate the developmental trajectory of children in the relevant studies.
- Evaluated children ≥ 24 months. If a broader age range is reported, we will extract and report age-specific findings separately.
- Conducted in countries classified as low- and - middle-income economies by the World Bank. The World Bank categorizes countries into four groups according to income/economy including low, lower-middle, upper-middle, and high income, and measures income using gross national income (GNI) per capita. Additional file (2) is attached for details on current criteria and a list of eligible countries.
- Had main study outcome as neurodevelopmental (cognitive, motor, and language domains); measured by a standardized assessment tool at two or more time points.
- For objective 2, mention at least one or more factors/variables of neurodevelopment (factors that influence developmental processes).
- Published in English or with an English translation as we lack access to scientific translation services.

### Exclusion criteria

- Review and cross-sectional studies, case series, and case reports will be excluded.

Data from gray literature, such as unpublished papers and sources not subject to peer review, will be excluded. Incorporating data from unpublished studies can introduce bias [17].

- Studies that did not use a standardized measurement tool for neurodevelopment or did not provide details on how neurodevelopment was measured will not be included.
- We will also exclude studies that do not report neurodevelopmental variables and studies failing to report separate findings between age groups.

The PECOST framework (Population /Participants, Exposure, Comparator, Outcomes, Setting/study design, and Time/date of publication) has been used to develop our review question. It will form the basis of the search for evidence (as indicated in Table 1).

### Population/Participants:

Studies examining children aged 0–24 months in LMICs will be reviewed. In case a wider age range is reported, we will extract and report age-specific findings. The inclusion of studies with longitudinal design will enable us to obtain their developmental pro le over time/developmental trajectory.

### Exposure condition:

We will include any variable that affects neurodevelopment specified in the included studies at different time points. They may include predictors, risk and protective factors such as infection, socio-economic factors including socio-economic status, quality of the child’s home environment, caregiver education and emotional wellbeing, and nutritional status among others.

For a study involving infections, it must have used a standard diagnostic method such as microscopy among others to be included.

### Comparator:

Studies that have compared neurodevelopmental outcomes between time points as well as variables of neurodevelopment at different time points.

### Outcomes:

The primary outcome of this systematic review will be the measurement of child neurodevelopment in LMICs (change in neurodevelopment between time points) and factors that influence neurodevelopmental processes.

### Setting and study design:

We will only include longitudinal and cohort studies conducted in LMICs as determined by the World Bank, 2024 [18].

### Timing/Date of Publication:

For timing, we will consider the different time points at which the neurodevelopment assessment was performed. For the neurodevelopment assessment to be considered, it should have been performed at two or more time points, between 0 and 24 months of the child’s age. Additionally, studies should have been published between 1990 to date.

### Language:

This systematic review will be limited to studies published in English and accessible in full text. We will only include studies published in English due to a lack of access to scientific translation services.

### Information Sources and Search Strategy

#### Information sources

Published articles on child neurodevelopment and associated variables will be searched from Psych INFO, PubMed, EMBASE, and Google Scholar databases. Additionally, reference lists of all selected studies that would have met our inclusion criteria will be hand-searched to check for additional potential articles to be considered. The search strategy will be developed by the review team. We will include all studies published from the year 1990 until the time the search is performed. Starting from the year 1990 will enable us to include even the initial longitudinal studies on child development conducted in LMICs for example the Pelotas (Brazil) birth cohort study conducted between 1982–1987 and published in 1990 [19].

### Search strategy

#### Search terms

Using the “all text” field and Boolean operators “OR” and “AND”, the search strategy will incorporate the following search terms: Trajectories, predictors, “child neurodevelopment”, “first 2 years” and “low-and- middle-income countries”. The specified terms and their associated synonyms will be used to create the search string (additional le 3).

#### Screening of studies

Studies will be included if they meet the specified inclusion criteria. All identified studies from our systematic search in the different databases; both abstracts and full texts will be imported to EndNote reference management software and duplicates removed. Two autonomous reviewers will screen articles based on the specified eligibility criteria by abstract, title, and full-text using EPPI Reviewer software. At abstract/ title screening, each of the reviewers will read the title and abstract to determine if it meets the inclusion criteria, and deliberate as follows; “No”, if the article fails to meet the inclusion criteria and thus should not be included, “Maybe” if there is insufficient information in the title or abstract for the reviewer to make an informed decision (a full-text screening will then be performed) and “Yes” if the article seems to meet the inclusion criteria and warrants further screening for full-text. We will obtain full texts for all titles/ abstracts that seem to meet the inclusion criteria. At full-text screening, all full-text articles of eligible studies will further be screened against the inclusion criteria. The two reviewers will be kept unaware of each other’s screening results during the process to ensure that all studies that qualify are included in the review [20].

Any disagreements between the two reviewers will be discussed and in case of further disagreement, a third reviewer will be involved as a tiebreaker A PRISMA flowchart will be developed to record the review process, including statistics of studies at each at each stage of the selection process along with justifications for not considering full-text articles.

#### Data extraction and management

Data from the final list of articles will be extracted by two reviewers autonomously using a pre-designed extraction form created in Microsoft Excel to collect study information. The form will be tested on five (5) articles and adjustments made if necessary. Any conflicts that emerge will be addressed by discussion, and further disagreement, if any, referred to a third reviewer as a tie-breaker. Prior to the extraction exercise, we will hold a meeting between the reviewers to ensure consistency. Data will be exported to Stata V.18 for meta-analysis.

##### Data items

Data items by the PRISMA-P will be extracted from the full texts of the included articles on the following;

- General information: the authors, publication year, article title, and country where the study was conducted.
- Study characteristics including study objectives, study design, sample size, name, and description of the neurodevelopmental assessment tool used.
- Participant characteristics: Timepoint (child’s chronological age at which assessment was done at the different visits), and targeted data/outcomes (neurodevelopment test performance scores and factors related to the test performance scores at the different time points i.e. predictors/risk factors/determinants/covariates).

#### Brief Overview of the Initial Findings from the electronic search of the PubMed database

Before planning a full systematic review, it is important to establish the extent and volume of evidence. We have thus listed five (5) potentially eligible studies from our electronic search of the PubMed database (Table 2). The studies were published between 2016 and 2023. Some trajectory patterns identified included consistently high scores, increasing scores, intermediate scores with early or late decline, and consistently low scores. Factors reported to discriminate between the trajectories include emotional or verbal responsivity of the caregiver, presence of stimulation and learning opportunities, the proportion of days a child experienced diarrhea, iron status, stunting, family socioeconomic status, and maternal depression.

#### Addressing Missing Data

Missing participant data can introduce meta-analysis bias [24]. Therefore, we will contact the authors of the respective studies to request any missing information. If the missing data is required for calculating effect size and is no longer available, we will exclude those studies however, they will be incorporated in the qualitative summary of eligible studies. No statistical models will be used to calculate missing data.

#### Critical Appraisal of Studies

##### Assessment of methodological quality (risk of bias) and grading strength of evidence

To reduce the risk of bias, two independent reviewers who did not participate in the screening process will critically appraise all selected studies for methodological quality and strength of evidence using the Grading of Recommendations Assessment, Development, and Evaluation (GRADE) system. The GRADE system is becoming popular as the preferable system for assessing the quality of evidence and strength of recommendations [16]. Based on the GRADE guidelines, the quality of outcomes will be classified as very low, low, moderate, or high-quality evidence.

We will also assess for potential reporting bias, particularly, publication bias via funnel plot asymmetry. Publication bias refers to the decision to publish or not publish study results which is influenced by the nature and direction of the results [25]. We will assess whether the sample of studies obtained is likely to be biased because of selective publication or identification. We will construct a funnel plot graph where we will plot each study effect size against its precision and use the symmetry of plots to detect the likelihood of publication bias among the included articles. We will adjust for publication bias using the trim and fill method. First, we will trim the data by eliminating studies starting with the least powerful until we achieve symmetry in the funnel plot and obtain a new pooled estimate from the remaining studies. Secondly, we will fill in the identified holes by reflecting the eliminated studies in the pooled estimate line.

#### Data synthesis and statistical analysis

##### Data synthesis for the systematic review

Data extracted from the included studies will be synthesized narratively by two reviewers, one will develop the synthesis and the other will check the findings. The review process will be demonstrated by a PRISMA flow chart. We will carry out a thematic analysis to summarize data on variables that affect neurodevelopment. Identified information from the selected studies will be coded, codes organized into sub-headings and descriptive categories, and the categories developed into analytic themes.

##### Data analysis for the meta-analysis

The level of heterogeneity that may exist among selected studies will determine if the data are amenable to a meta-analysis being performed that is; two (2) [26] or more studies with sufficient homogeneity in terms of the same neurodevelopment measurement tool used, the same sample characteristics, and methods (setting and length of follow-up). Potential sources of heterogeneity include neurodevelopmental domains assessed, measurement tools used, and sample size.

If it is possible to perform a meta-analysis, STATA 18. will be employed for data analysis. Descriptive statistics including standard mean differences, Standard deviations, and 95% confidence intervals (CIs) will be utilized to summarize the findings. The standard means differences will be interpreted as per Cohen’s guidelines [27]. Effect sizes of 0.2, 0.5, and 0.8 will be considered small, medium, and large respectively. A random effects model is advisable when heterogeneity is anticipated [28]. Given that we expect samples to vary in number and characteristics, and varying assessment measures of neurodevelopment to be used, we shall calculate pooled estimates using a random effects model to control for the heterogeneity. Heterogeneity between studies will be assessed using forest plots, p values, and I-squared statistics index values with 95% CIs. I-squared statistic percentage heterogeneity values will be interpreted as follows; 0% as no observed heterogeneity, 25% as low, 50% as moderate, and ≤75% as high [29].

#### Sensitivity analysis

Provided adequate data exists, we shall perform sensitivity analysis to establish the robustness of the meta-analysis. Specifically, we shall systematically exclude studies, one at a time, and assess the consistency of the overall effect [30].

## Discussion and Outcomes

This review will identify key factors affecting child neurodevelopment in LMICs, and characterize the developmental profile of children over the first 2 years of life thereby providing a better understanding of the early development of children in this region. Findings from the study will also support the design of more focused and effective interventions and policies for enhancing child development in LMICs.

### Anticipated limitations

The inclusion of only English publications may introduce bias whereby some relevant data may be missed. Some studies may be excluded because of unavailable data.

## Figures and Tables

**Table 1:** Summary of the PECOST Framework as implemented in the proposed protocol

PECOST Concepts	
Population	Children aged 0–24 months
Exposure	Any variable that affects neurodevelopment specified in the included studies
Comparator	Studies that have compared neurodevelopmental outcomes between time points as well as variables that affect neurodevelopment at different time points
Outcome(s)	Change in neurodevelopment scores between time points and factors that affect neurodevelopment
Setting	Low-and -Middle-Income Countries as classified as by the World Bank, 2024
Timing/Date of Publication	Reported outcomes at two or more time points and were published from 1990(initial studies on child neurodevelopment in LMICs were published about this time) to date.

**Table 2:** Preliminary results: Characteristics of five (5) potentially eligible studies

Study	Location	Population	Sample size	Outcome Measure(s)	Ref. No
Koshy et al., (2021)	India	Children	251	Bayley Scales of Infant and Toddler Development-third edition	[12]
McCormick et al., (2020)	8 LMICs: Bangladesh, Brazil, Peru, India, Nepal, Pakistan, South Africa and Tanzania	Children	835	Bayley Scales of Infant and Toddler Development-third edition	[14]
McCann et al., (2023)	Gambia	Children	179	Mullen Scales of Early Learning	[21]
Jiang et al., (2017)	Bangladesh	Children	422	Bayley Scales of Infant and Toddler Development-third edition	[22]
Pereira et al., (2016)	Southern Brazil	Children	49	Alberta Infant Motor Scale Bayley Scales of Infant Development (Mental Development Scale)	[23]

## Abbreviations

CIs: Confidence Intervals
GNI: Gross National Income
GRADE: Grading, Recommendation, Assessment, Development and Evaluation
LMICs: Low-and-Middle Income Countries
MeSH: Medical Subject Headings
PRISMA-P: Preferred Reporting Items for Systematic Reviews and Meta-Analysis Protocols
PROSPERO: International Prospective Register of Systematic Reviews
RoB: Risk of Bias
